# Epilepsy and Education in developing countries: a survey of school teachers’ knowledge about epilepsy and their attitude towards students with epilepsy in Northwestern Nigeria

**DOI:** 10.11604/pamj.2014.18.255.3607

**Published:** 2014-07-27

**Authors:** Lukman Femi Owolabi, Naziru Muhammad Shehu, Shakirah Desola Owolabi

**Affiliations:** 1 Neurology unit, Department of Medicine, Aminu Kano Teaching Hospital, Nigeria; 2 Department of Psychiatry, Aminu Kano Teaching Hospital, Kano, Nigeria, Bayero university, Kano, Nigeria

**Keywords:** Epilepsy, student, knowledge, attitude

## Abstract

**Introduction:**

Students living with epilepsy still find themselves confronted with social barriers that prevent them from academic achievements.

**Objective:**

The aim of this study was to evaluate knowledge and attitudes of school teachers and to determine the factors associated with good knowledge and positive attitude among nursery, primary and secondary school teachers in Kano, Northwestern Nigeria.

**Methods:**

A 20-item validated semi-structured questionnaire was self-administered to 200 teachers, selected through multistage sampling technique, in Kano, Northwestern Nigeria.

**Results:**

The respondents comprised 124 (62%) males and 76(38%) females. Their age ranged between 17 and 55 with a median age of 26 years. All the respondents have heard or read about epilepsy, 13% have had students with epilepsy in their classes before, fifty one (25.5%) of them would object to having an epileptic child in their class. To one hundred and seventy one (85.5%) all seizures were convulsive, 81% of them admitted to not havingadequate knowledge of the initial procedure should their student develop epileptic seizure, 60% believed that epileptic student should be separated from non-epileptic students in the classroom and 44.5% of them preferred alternative medicine. Overall, fifty nine (29.5%) teachers had fair to good knowledge about epilepsy. and one hundred and sixty four (82%) had positive attitude to students with epilepsy. Statistically significant association was found between level of education attained by the respondents and good knowledge about epilepsy as well as between female gender and good attitude. However, none of the variables was statistically significant predictor of knowledge or attitude among the respondents.

**Conclusion:**

There was low level of knowledge and misconceptions about epilepsy but fair attitude towards epileptic students in Northwestern Nigeria. Higher level of education of the teachers was significantly associated with good attitude.

## Introduction

In ancient times, persons with epilepsy were perceived as “possessed”, and up till now a large number of people still hold this belief as well as the belief that epilepsy is contagious [1]. Despite various educational programs implemented in the general public the misperceptions of epilepsy continue and have not improved much over time [2]. In many parts of Africa there is still a lot of superstition about epilepsy [3].

Apart from the disability associated with repeated seizures or side-effects of antiepileptic medications, the social stigma, arising from knowledge about the disease, is often a major handicap to people living with this condition [4]. Students with epilepsy still find themselves confronted with social barriers that prevent them from academic achievements, in addition to the limitation that the disease itself has already placed on them [5].

Report from a study conducted among the general populace in Kano showed low level of knowledge and high level of misconceptions among the respondents [6].

School represents a significant component of life for all children. It is a place where they spend a large amount of their time during a critical period of social, psychological, and physical development. Moreover, because success in school is so important to success in adult roles, school experiences are a key factor in students; current and future quality of life [7–9].

Similarly, school teachers play a central role in determining access to education of the students living with epilepsy and may mediate epilepsy-associated stigma [10]. Besides, attitude of school teachers towards epilepsy is likely to have an important impact in the schooling of children with epilepsy. Thus, in order to ensure a holistic approach to school education of students living with epilepsy, a clear understanding of their teacher's knowledge about epilepsy and their attitudes towards the disease will be worthwhile.

The aim of this study was to evaluate knowledge and attitudes of school teachers and also determine the factors associated with good knowledge and positive attitude among nursery, primary and secondary school teachers in Kano, Northwestern Nigeria.

## Methods

### Survey setting

The study was conducted in urban area of Kano state in northwestern Nigeria. Apart from being a centre of commerce, the city is considered a region with high level of educational activities. There are several private and public primary and secondary schools in the city. Using a multi stage sampling technique, three local government areas (Fagge, Tarauni and Nasarawa) were selected out of the eight local government areas (LGA) in Kano city. The study was conducted in twenty randomly selected schools in the three LGAs. Study design The study was a cross-sectional and descriptive in design.

### Study instrument

A 20-stem semi-structured questionnaire which was previously validated to evaluate knowledge, attitude and beliefs among adults in Kano [6] and elsewhere, [11] with some modification, was used. The study instrument, designed in English language, was pretested in a school in Taraunilocal government area of Kano State and appropriate adjustment was made subsequently. The questionnaire was structured to elicit demographic information and to test the knowledge of the teachers of the cause of epilepsy, the first-aid management of an epileptic attack and the attitude towards the student or pupil with epilepsy.

The questionnaire was in three parts; the first part was about personal demography of the respondent, the second part explores awareness of existence of epilepsy in the community, knowledge of its causal factors, knowledge of whether the disease is transmissible or not, methods of prevention, first aid when a seizure attack occurs as well as preferred treatment option and the third part enquired about the attitudes, perception and behavior of the respondents towards epileptic students. Thirty responses were expected in the part assessing knowledge of the respondents.

Regarding the questions about the initial procedures in attending to student during a seizure, we considered as main correct responses in affirmative; to protect and to turn the head to one side in order to permit the saliva flow, to keep ventilation on and to stay near the subject until the end of the seizure, avoiding any harmful situations. We considered as less important, correct initial procedures, acts like taking off clothes and removing nearby objects [12]. Attitudes such as fear, avoidance, anger, suspicion, mistrust, hostility were regarded as negative attitudes whereas sympathetic attitude, willingness to care for epileptics and tolerance were considered positive attitudes.[6] Three university undergraduates who were trained to administer the questionnaire collected the data. Ethical approval was obtained from all the schools where the study was conducted.

### Data analysis

Analysis of data was carried out using the “Statistical Package for Social Sciences” (SPSS) program for Windows version 16.0 (SPSS Inc., Chicago, IL). Continuous variables such as age were expressed as the range and median, whereas categorical variables were presented as frequencies (%). Chi-square test was used to examine the association between responses and each demographic variable in a Univariate analysis. Significance level was set at P < 0.05. The variables that were statistically significant on Univariate analysis were subjected to multiple logistic regression to identify independent predictors of knowledge or attitude.

Correct responses to questions on knowledge were given a score of one point if answered correctly. No point was given for wrong answers. Based on this grading, a total of 30 points were allocated to the section on knowledge about epilepsy, such that those who scored 21-30 points were considered as having good knowledge, 21-30 points had fair knowledge, while a score of 0-10 points was considered as representing poor knowledge or a score of > 10 (fair to good knowledge and = 10 (poor knowledge). Attitudes such as fear, avoidance, anger, suspicion, mistrust, hostility were considered negative, whereas sympathetic attitude, willingness to care for epileptics and tolerance were considered positive attitudes [6].

## Results

Out of 246 questionnaires distributed a total of 227 were completely filled and returned, giving response rate of 92.3%. Out of the 227 questionnaire received, 200 were suitable for analysis. There were 124 (62%) males and 76(38%) female respondents. Their age ranged between 17 and 55 with a median age of 26 years. Majority (76%) of the respondents were aged between 21 and 40 years. One hundred and seventy one (85%) were Muslims and twenty nine (14.5%) were Christians. The Hausa-Fulani ethnic group constituted 64.5% of respondents, Yoruba 10%, Igbo 5.0% and other minority Nigerian tribes were 22.5%. Out of the schools selected nine (45%) were public schools and eleven (55%) were private owned schools. Fourteen (70%) were nursery and primary schools, six (30%) were secondary schools. About 22% of respondents had secondary school education, 57.5% were graduates of college of education, and 28% had university or polytechnic education. One hundred and fifty six (78%) of the respondents had had 1-5 years of teaching experience. All the respondents have heard or read about epilepsy, 13% have had students with epilepsy in their classes before, 32% had seen at least an incident of epileptic seizure and 2% had at least a relative with epilepsy. Fifty one (25.5%) would object to having an epileptic child in their class. One hundred and seventy one (85.5%) thought that all seizures were convulsive. Out of the respondents, one hundred and sixty two (81%) admitted they did not have detailed knowledge of the initial procedure should their student develop epileptic seizure.

Table 1 showed the teachers’ response to questions evaluating their knowledge about epilepsy. Responses of the teachers to questions evaluating their attitude towards students with epilepsy are as outlined in Table 2. 83% of the respondents felt there was discrimination against epileptic students and 85.5% of them believed the discrimination was unjustified. One hundred and twenty (60%) respondents believed that epileptic student should be separated from non-epileptic students in the classroom (Table 3). Regarding treatment options, the respondents preferred, 44.5% of them preferred alternative medicine (Table 4).

**Table 1:** Response of the teachers to questions evaluating their knowledge about epilepsy.

Result	Yes	No
**What do you think an epileptic attack is?**		
An abnormal electrical discharge in the brain	84	116
Demonic (Jins) possession	129	71
An abnormal movement	91	109
What types of seizures exist?		
Generalized tonic-clonic seizures	178	22
Simple partial seizures	3	197
Complex partial seizures	2	198
Atonic seizures	80	120
Absence seizures	21	179
Neither here nor there (unclassified)	23	177
**What do you think causes epilepsy?**		
Demonic possession	152	48
Head injury	100	100
Brain tumor/cancer	131	69
Divine punishment	40	160
Witchcraft	80	120
Alcohol withdrawal or heavy drinking	45	155
Stroke	63	137
Hereditary	114	86
High fever	100	100
Eating forbidden food	20	180
Brain infection	100	100
Birth trauma	97	103
Don't know	43	157
Sometimes, the cause may not be identified	88	112
Is epilepsy contagious?	120	80
**What should be done during a seizure?**		
Place in semi-prone position to prevent choking	87	20
Place an object in the mouth of the student to prevent the mouth from closing	180	
Make sure his/her saliva does not touch the other students	122	78
Restrain and perform chest compressions (CPR)	57	143
Take actions to prevent injury during an episode	89	111
Run out of the class to call the other teachers	18	182

**Table 2:** Response of the teachers to questions evaluating their attitude towards students with epilepsy.

Attitude	Yes	%
Tolerance	21	10.5
Fear	0	0
Hostility	0	0
Kindness	98	49.0
Sympathy	40	20
Suspicion	0	0
Avoidance	22	11
Indifference	19	9.5

**Table 3:** Response to other questions on attitude.

Result	Yes	No
Do you belief students with epilepsy always have behavior pattern different from non- epileptic students?	178	22
Are students with epilepsy academic performances always poor?	94	106
Can students with epilepsy have excellent performance?	81	119
Are teachers often informed by parents about their child illness?	40	160
Do you think epileptic student should be separated from non-epileptic students?	120	80
Can epilepsy be cured?	60	140

**Table 4:** What treatment option would you prefer for epileptic students?

Response	No	%
Orthodox medicine	111	55.5
Traditional medicine	29	14.5
Spiritual healing	60	30

Using the score for knowledge about epilepsy, overall, only fifty nine (29.5%) teachers had fair to good knowledge about epilepsy. On the contrary, one hundred and sixty four (82%) had positive attitude to students with epilepsy. Table 5 and Table 6 highlights the association between demographic variables, knowledge about epilepsy and attitude respectively. However, on multiple logistic regression analysis none of the variables appeared as an independent predictor of knowledge about epilepsy or attitude towards students with epilepsy.

**Table 5:** Relationship between demographic variables and knowledge about epilepsy.

Variable	Number of those with fair to good knowledge/total number	Odd ratio (95% CI)	P value
**School type**			
Private	35/112	1.2(0.63-2.35)	0.370
Public	24/88		
**Level of education of the respondent**			
Tertiary	53/161	2.7((1.01-7.68)	0.030*
Secondary	6/39		
**No of years of teaching experience(Years)**			
>5	15/44	1.3(0.61-2.84)	0.450
<5	44/156		
**Gender of the respondents**			
Female	25/76	1.3(0.67-2.53)	0.41
Male	34/124		

* P value is statistically significant

**Table 6:** Relationship between demographic variable and positive attitude towards student with epilepsy.

Variable	Number of respondent with positive attitude/total number	Odd ratio (95% CI)	P value
**School type**			
Private	90/112	0.8(0.25-7.16)	0.495
Public	74/88		
**Level of education of the respondent**			
Tertiary	131/161	0.8(0.35-1.71)	0.483
Secondary	33/39		
**No of years of teaching experience(Years)**			
>5	38/44	1.5(0.55-4.38)	0.394
<5	126/156		
**Gender of the respondents**			
Female	69/76	3(1.17-8.04)	0.011*
Male	95/124		

* P value is statistically significant.

## Discussion

Epilepsy, despite being a common neurological disorder since the ancient times, has eluded the basic understanding of many people including the teachers who should play a pivotal role in disseminating basic knowledge of epilepsy to the students. Our study showed that the knowledge of teachers about epilepsy was generally poor. This finding agrees with the reports elsewhere [13–15]. Several teachers have little or no knowledge about the basic manifestations as well as types of epileptic seizure, many of them believed that all epileptic seizures are convulsive. This finding, which is in agreement with other reports, [16–18] reflects dearth of specific training in respect of epilepsy. Similarly, the finding has a great implication for in-school recognition of seizure disorder;many non- convulsive epilepsy could be missed or mistaken for something else by the teacher resulting in non or delayed notification of parent or guardian of such student about his or her illness.

In this study, a large number of the teachers believed that epilepsy is caused by demonic possession and evil spirit, similar finding was reported in a study conducted on the general populace [6]. Unlike in the developed countries, this belief is still prevalent in the developing countries of the world. Reports from Zimbabwe [19], Ethiopia [18] and Brazil [13] from studies conducted on teachers, were also in agreement with our finding. Similarly, population study in Hong Kong revealed that 2% of respondents believed that epileptic disorder is related to demonic possession [20]. In some other reports, 5.2% of India's population, [21] 6.3% of Sri Lanka's inhabitants, [22] 30% of rural Ethiopians,[23] and 27.9% in a Ghanaian study[24] also have the same belief. Epilepsy particularly when manifested as generalized tonic-clonic seizure provokes strong and ambivalent feelings in those witnessing it. An atmosphere of fear, shame and mysticism surrounds epilepsy since ancient times even in our days in many non-Western cultures. This negative belief has bearing on the attitude and behavior exhibited by the teachers towards students with epilepsy and these popular notions continue to degrade the educational attainment and quality of life of students with epileptic seizures.

One of the most important revelations of the study is the belief, by some of the teachers, that epilepsy is contagious, a belief that reflects poor understanding of the disorder. This belief may partly explain why some of the respondent may be opposed to having a child with epilepsy in their class, it may also partly account for their support for separation of non-epileptic from children with epilepsy in the classroom as seen in this study.

It is interesting to note that many of the teachers were not familiar with the initial procedures and first aid in attending to a person during a seizure. In this study, the initial procedures adopted by some the teachers such as putting an object in the mouth of the child during an episode of epileptic seizure and making sure the saliva from the child does not touch another student were grossly inappropriate. Some of the wrong procedures, which are potentially harmful, are still related to mythical concepts [12, 25].

In the present study, 82% of the teachers had a positive attitude to students with epilepsy. This finding, which is similar to the result obtained from the survey of general population in Kano city, agrees with the report from western countries [9, 26, 27]. Attitude researchers have indicated that in the course of time, attitudes toward people with epilepsy have consistently improved [26, 27].

Nonetheless, in another but similar study using an indirect measure, a less positive attitude than was found in the present study was recorded [28]. Our result also showed some improvement on reports from South-southern Nigerian obtained about thirty years ago which showed widespread negative attitude toward epileptic patients in the Nigerian society [29]. On the whole, the general attitude toward epileptic students is positive, however, looking at composite attitude individually, it is noteworthy that a number of the respondents still entertained avoidance and fear for students with epilepsy. As a result, epileptic students suffer untold social deprivations and discrimination in education.

Also of note is the preference, of about 51% of the teachers, for spiritual healing and traditional treatment. In a study to evaluate the attitude of to traditional and spiritual healing in Tanzania, almost half of the interviewed people believe that traditional and spiritual way of treatment of epilepsy could help treat people living with epilepsy [30]. This finding probably stemmed from the belief that epilepsy is caused by evil spirit and demonic possession and hence should be treated as such. Cultural beliefs and misconceptions about epilepsy often influence the care-seeking behavior in Africa, consequently, a significant proportion of Africans seek complementary and alternative means of treatment.

Higher level of education attained by the teacher was found to be significantly associated with good fair to good knowledge and female gender was found to be associated with good attitude in our study. Our finding agrees with result from community study in Kano [6] as well as report from similar survey elsewhere [24]. However, on adjustment for the other demographic variables none of these factors emerged as an independent predictor of either good knowledge about epilepsy or positive attitude towards students with epilepsy.

The attitude toward student with epilepsy may not be unconnected with the degree of knowledge of the condition [31, 32]. Teachers play a vital role in educational and psychosocial development of students as a large part of their developing life is spent at school, thus, a better educative structured program is desirable for these professionals with the view to reducing the myths and fears about epilepsy. In the long run, this might help reduce prejudice against students with epilepsy and increase the acceptance of epileptic individuals into the classroom. It is hoped that our finding would be found useful by the policy makers on education in the design or redesign of educational curriculum. Incorporationof education on epilepsy and similar conditions in will be worthwhile as this would make education less stressful for students living with epilepsy by eliminating negative social and psychological consequences such as fear, humiliation, and limitations in their social interactions in and outside school environment. Moreover, this information may be used to target and develop evidence-based interventions for teachers in schools. However, such intervention must take into account the belief, attitude and value systems of the school teachers and address misconceptions about epilepsy

## Conclusion

There was low level of knowledge and misconceptions about epilepsy but fair attitude towards epileptic students in Northwestern Nigeria. Higher educational attainment of the teachers was significantly associated with good attitude about epilepsy. The need for a well-structured educational and evidence-based intervention for teachers about epilepsy cannot be overemphasized.
